# Supplementary figures and images for: Blockade of IKK signaling induces RIPK1-independent apoptosis in human macrophages
Source: PLoS Pathog. 2024 Aug 26;20(8):e1012469. doi: 10.1371/journal.ppat.1012469 (PMC11407650; doi:10.1371/journal.ppat.1012469)

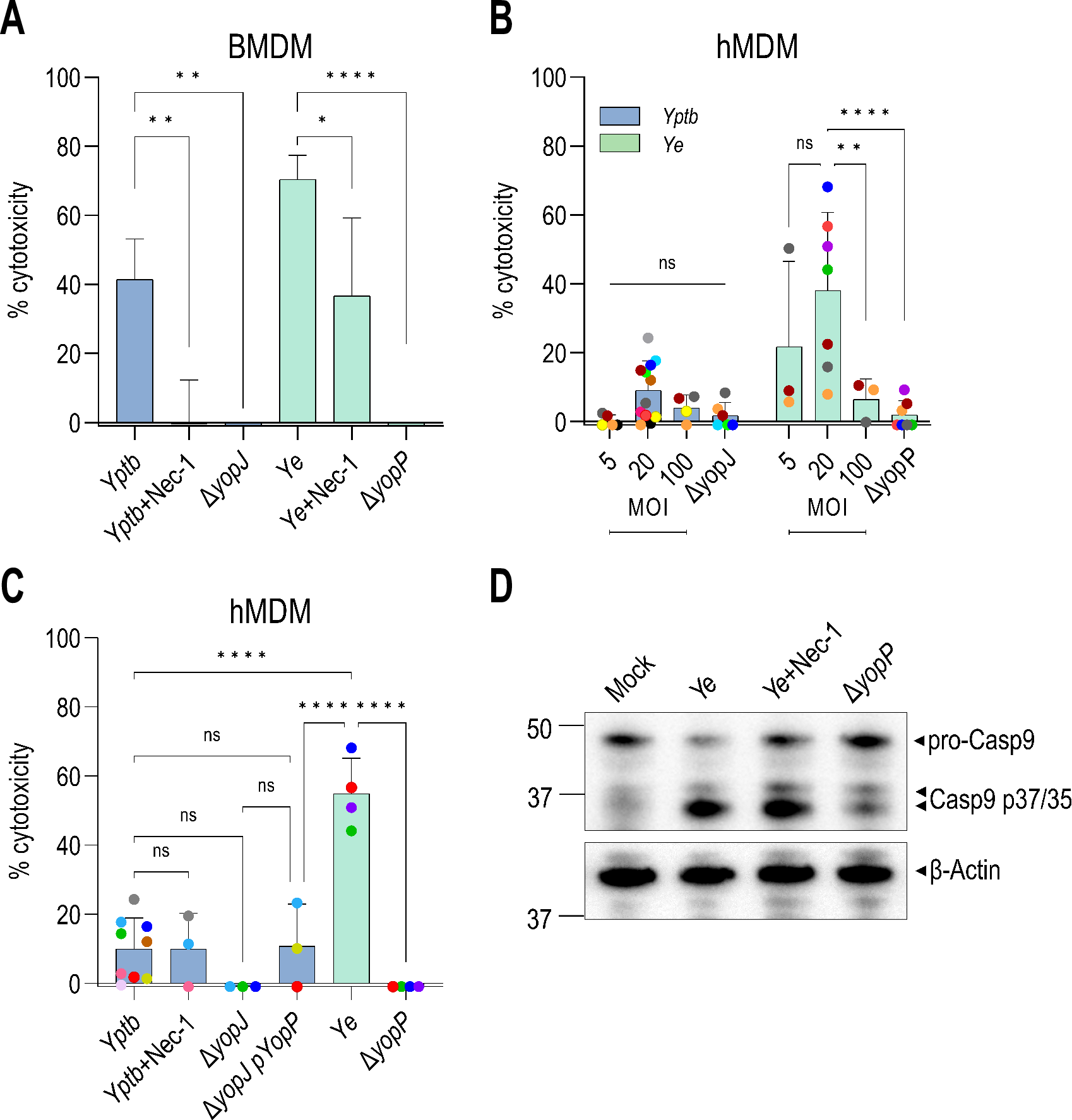

Supplement: S1 Fig — Cells were pre-treated with Nec-1 and then infected with the following strains of Yersinia: WT Y. pseudotuberculosis (Yptb), ΔyopJ Yptb, ΔyopJ pYopP Yptb, WT Y. enterocolitica (Ye), or ΔyopP Ye. Cytotoxicity was measured by LDH release. (A) BMDMs were infected for 4–6 h at MOI 20. N = 3. (B–C) hMDMs were infected for 16–22 h. Each data point represents the mean of triplicate wells for each of 7–12 different human donor hMDMs. (D) Immunoblot analysis was performed on hMDM lysates for caspase-9 and β-actin. Representative of 2–3 independent experiments. ns, not significant, *p < 0.05, **p < 0.01, ***p < 0.001, ****p < 0.0001 by (A) Šídák’s or (B) Tukey’s multiple comparisons test. Graphs depict mean + SD. (TIF) [file ppat.1012469.s001.tif]

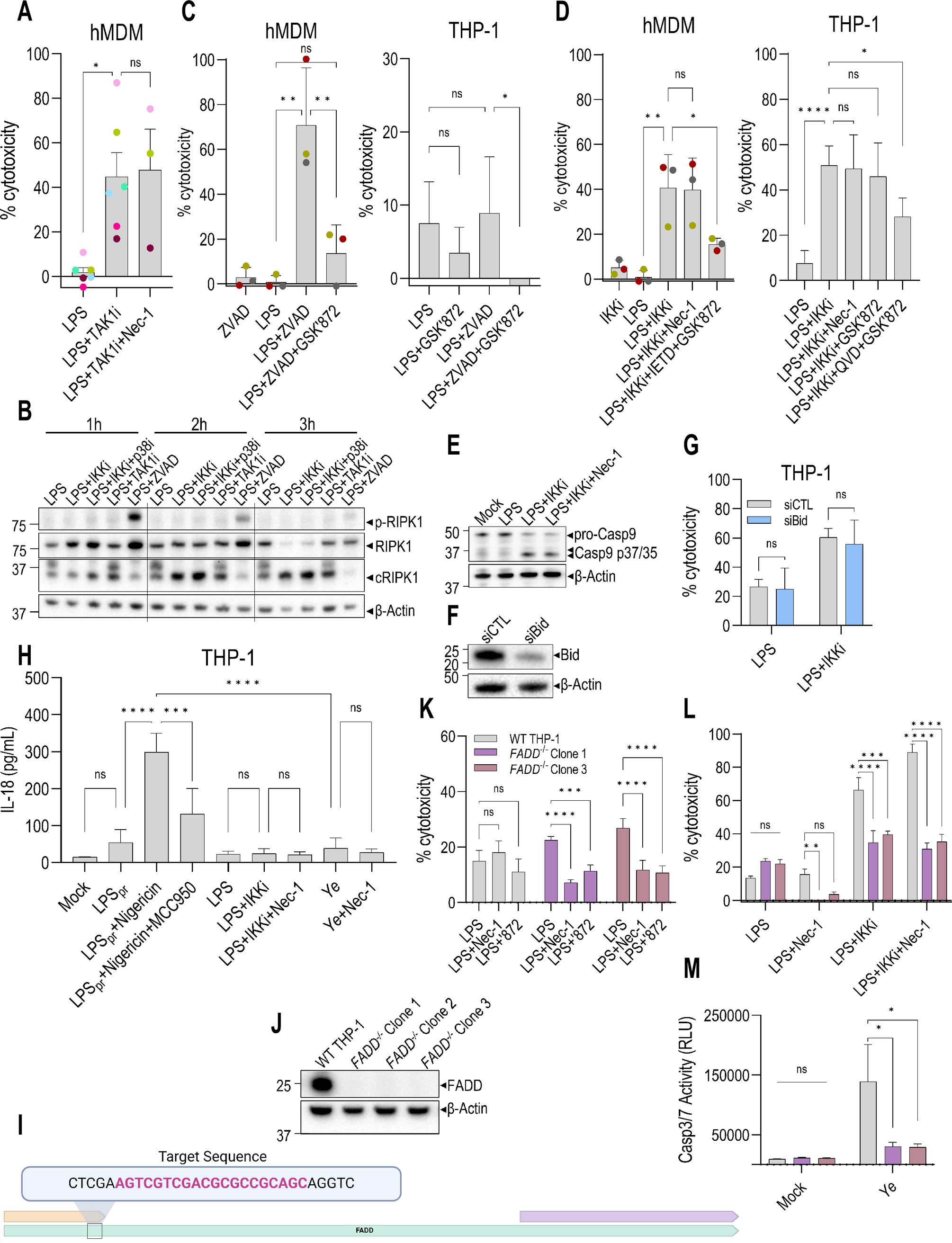

Supplement: S2 Fig — Cells were pre-treated with inhibitors and then stimulated with LPS or infected with Ye. (A) hMDM cytotoxicity was measured by LDH release 5–7 h after stimulation, N = 3–6. (B) Immunoblot analysis was performed on hMDM lysates at various time points for phospho-RIPK1 (S166), RIPK1, and β-Actin. Representative of 2–4 independent experiments. (C,D) hMDM cytotoxicity was measured by LDH release after 10 h of stimulation. N = 3. THP-1 cell cytotoxicity was measured by LDH release 17–24 h after stimulation. N = 3–7. (E) Immunoblot analysis was performed on hMDM lysates for caspase-9 and β-Actin. Representative of 2–3 independent experiments. (F–G) THP-1 macrophages were transfected with siRNA specific for BID (siBid) or scrambled siRNA (siCTL) for 4 days, pre-treated with IKKi, and then stimulated with LPS. (F) Immunoblot analysis was performed on lysates 22 h after mock-treatment for BID and β-Actin. Representative of 4 independent experiments. (G) Cytotoxicity was measured by LDH release 22 h after stimulation. N = 4. (H) Depending on condition, THP-1 macrophages were LPS-primed (Lpr), pre-treated with IKKi, Nec-1, and/or MCC950, and then stimulated with LPS or Nigericin or infected with WT Ye for 23–24 h. IL-18 levels were measured by ELISA in the supernatant. N = 3. (I–J) Three independent FADD-/- THP-1 single-cell clonal cell lines were generated with CRISPR-Cas9. (I) Schematic representation of the FADD gene with exons (arrows), created using Benchling and BioRender. gRNA target sequence is highlighted in pink text. (J) Immunoblot analysis was performed on WT and FADD-/- THP-1 cell lysates for FADD and β-actin. (K,L) WT and FADD-/- THP-1 macrophages were pre-treated with inhibitors and then stimulated with LPS. Cytotoxicity was measured by LDH release 24–25 h post-stimulation. Representative of two independent experiments. (M) WT and FADD-/- THP-1 macrophages were infected with Ye. Caspase-3/7 activity was detected and quantified by Caspase-Glo 3/7 24–25 h post-inf [file ppat.1012469.s002.tif]

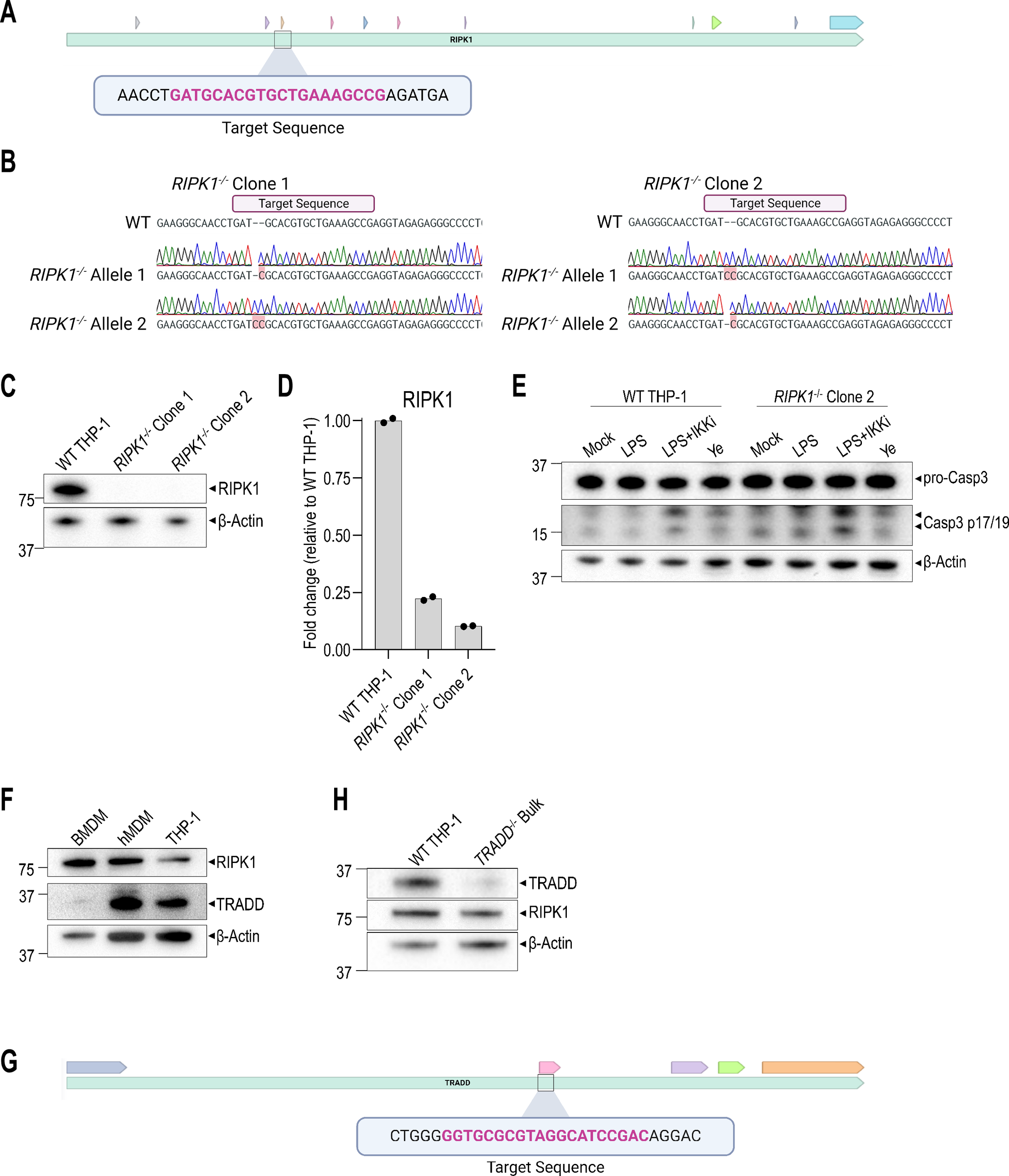

Supplement: S3 Fig — Two independent RIPK1-/- single-cell clonal cell lines were generated with CRISPR-Cas9. (A) Schematic representation of the RIPK1 gene with exons (arrows), created using Benchling and BioRender. gRNA target sequence is highlighted in pink text. (B) Sequence alignments of WT THP-1 and RIPK1-/- Clones 1 and 2 are shown for both alleles. Graphic was created using Benchling and BioRender. Red highlighting represents the mutated region. (C) Immunoblot analysis was performed on WT and RIPK1-/- THP-1 cell lysates for RIPK1 and β-actin. (D) RT-qPCR was performed on WT and RIPK1-/- THP-1 cell lysates for RIPK1 expression relative to HPRT. (E) Immunoblot analysis was performed on WT and RIPK1-/- THP-1 cell lysates 5–6 h after stimulation or infection for caspase-3 and β-actin. Representative of 2–3 independent experiments. (F) Immunoblot analysis was performed on WT murine BMDM, hMDM, and THP-1 cell lysates for RIPK1, TRADD, and β-actin. (G–H) Bulk TRADD-/- THP-1 macrophages were generated with CRISPR-Cas9. (G) Schematic representation of the TRADD gene with exons (arrows), created using Benchling and BioRender. gRNA target sequence is highlighted in pink text. (H) Immunoblot analysis was performed on WT and bulk TRADD-/- THP-1 cell lysates for TRADD and β-actin. (TIF) [file ppat.1012469.s003.tif]

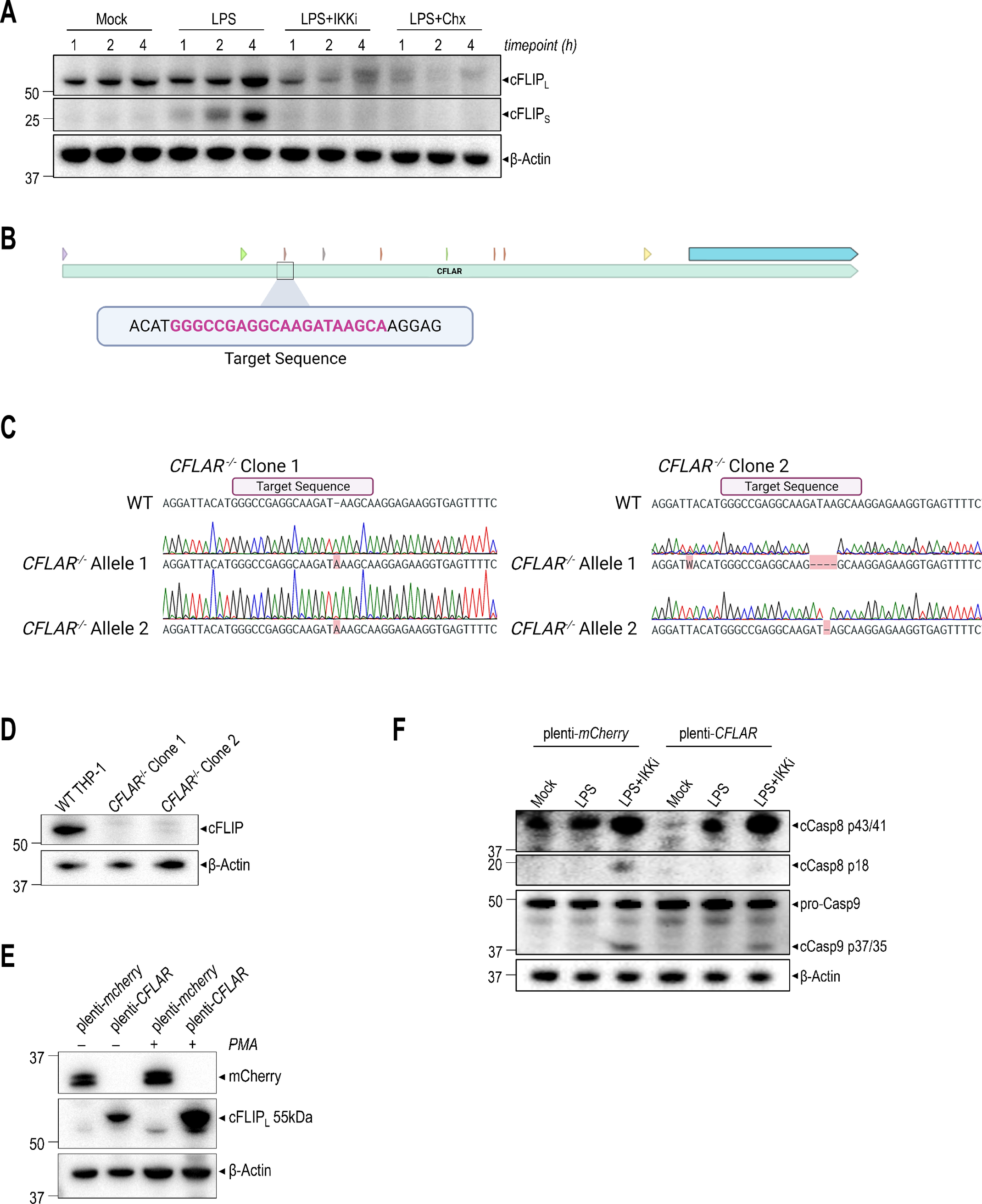

Supplement: S4 Fig — (A) hMDMs were pre-treated with IKKi or Chx and then stimulated with LPS. Immunoblot analysis was performed on lysates at various time points for cFLIP and β-actin. Representative of 2 independent experiments. (B–D) 2 independent CFLAR-/- single-cell clonal cell lines were generated with CRISPR-Cas9. (B) Schematic representation of the CFLAR gene with exons (arrows), created using Benchling and BioRender. gRNA target sequence is highlighted in pink text. (C) Sequence alignments of WT THP-1 and CFLAR-/- Clones 1 and 2 are shown for both alleles. Red highlighting represents the mutated region. (D) Immunoblot analysis was performed on WT and CFLAR-/- THP-1 cell lysates for cFLIP and β-actin. (E) Immunoblot analysis was performed for cFLIP on lysates from plenti-mCherry and plenti-CFLAR stably-overexpressing THP-1 monocytes and PMA-differentiated macrophages. (F) plenti-mCherry and plenti-CFLAR stably-overexpressing THP-1 macrophages were pre-treated with IKKi and then stimulated with LPS. Immunoblot analysis was performed on lysates 5 h after stimulation for cleaved caspase-8, caspase-9, and β-actin, representative of 2–3 independent experiments. (TIF) [file ppat.1012469.s004.tif]

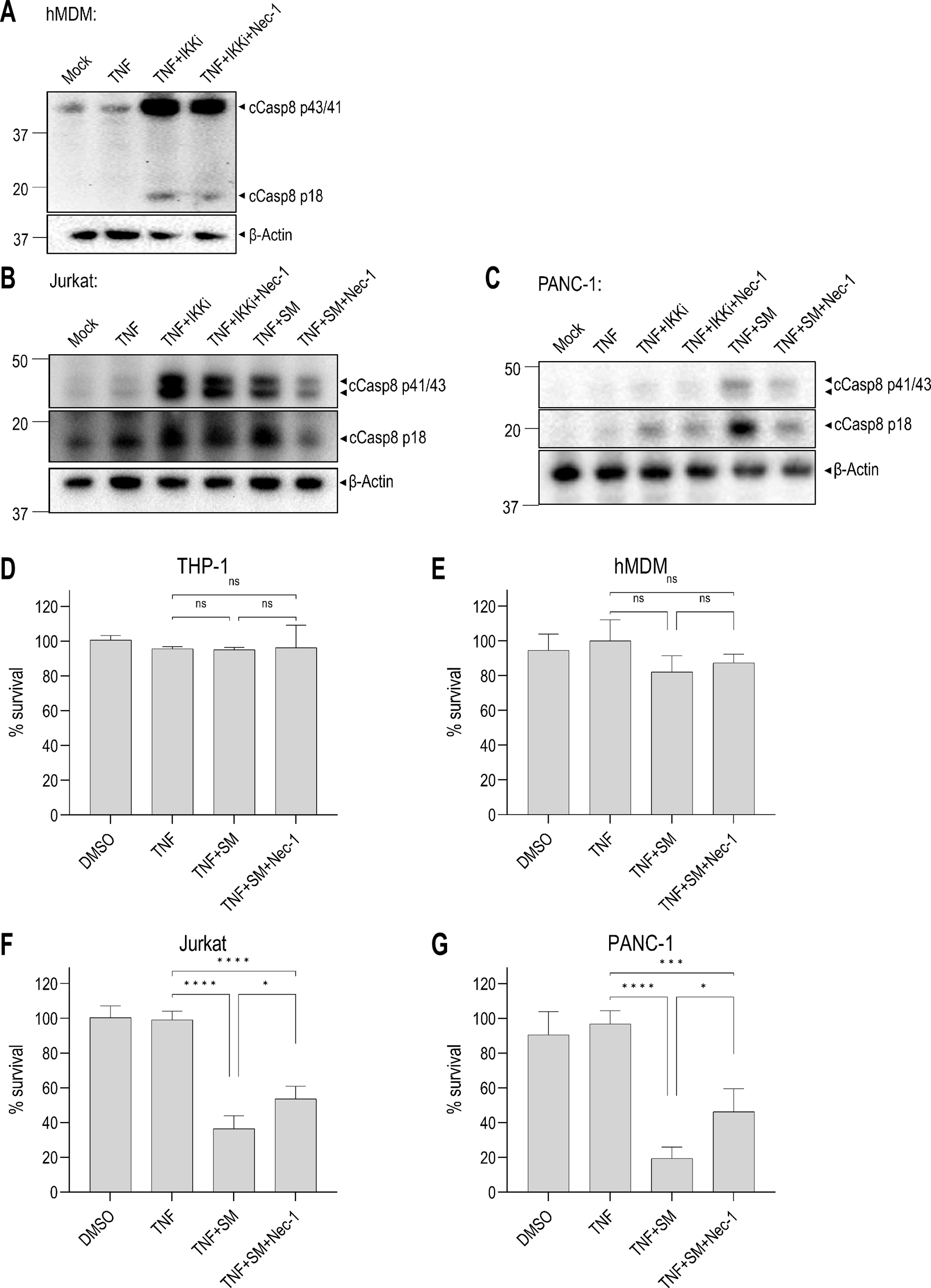

Supplement: S5 Fig — Cells were pre-treated with inhibitors or vehicle control (DMSO) and then stimulated with TNF. (A–C) Immunoblot analysis was performed on lysates 5–6 h after stimulation for cleaved caspase-8 and β-actin in the following cell types, representative of 2–3 independent experiments: (A) hMDMs, (B) Jurkat cells, and (C) PANC-1 cells. (D–G) Viability was measured by ATP signal in the following cell types: (D) THP-1 macrophages, 22–25 h, N = 4, (E) hMDMs, 5–6 h, N = 3, (F) Jurkat cells, 12–15 h, N = 4, and (G) PANC-1 cells, 14–16 h, N = 4. ns, not significant, *p < 0.05, **p < 0.01, ***p < 0.001, ****p < 0.0001 by Tukey’s multiple comparisons test. Graphs depict mean + SD. (TIF) [file ppat.1012469.s005.tif]
