# Supplementary material for: Blockade of IKK signaling induces RIPK1-independent apoptosis in human macrophages
Source: PLoS Pathog. 2024 Aug 26;20(8):e1012469. doi: 10.1371/journal.ppat.1012469 (PMC11407650; doi:10.1371/journal.ppat.1012469)
Supplement: Attachment — Submitted filename: Response to Reviewers - FINAL.pdf [file ppat.1012469.s007.pdf]

Response to Reviewers

Thank you for the opportunity to submit our revised manuscript: "Blockade of IKK signaling induces RIPK1-independent apoptosis in human macrophages" (Manuscript ID: PPATHOGENS-D-23-01101). We greatly appreciate the Reviewers' constructive feedback on our original submission. We are also grateful for the editorial summary of essential revisions. To address the Reviewers' comments, we have performed extensive additional experiments and provided additional discussion. We believe that our manuscript and its conclusions have been strengthened substantially by these new data and modifications to the manuscript. We provide point-by point responses to their comments below.

Reviewer #1:

Overall summary: *In the manuscript by Nataraj et al, the authors discovered that in human cells RIPK1 is not required for the induction of apoptosis triggered by IKK inhibition, either pharmacologic or by the Yersinia virulence factor YopJ. Indeed, they show that, unlike in mouse cells, in human cells, including human monocyte-derived macrophages and THP-1, Yersinia infection or LPS treatment combined with IKK pharmacologic inhibition induce RIPK1-independent apoptosis. Of note, neither the kinase nor the scaffolding activity of RIPK1 is required for apoptosis induction in these settings. Instead, IKK inhibition-mediated cell death could be partially rescue by cFLIP overexpression, consistently with the fact that IKK inhibition leads to cFLIP downregulation.*

Over the past decade, mouse studies have highlighted the role of RIPK1 kinase activity in a number of cell death-driven inflammatory pathologies, including Yersinia infection, Sharpin mutant-driven dermatitis and TNF-driven lethal sepsis. However, it is still not clear the contribution of RIPK1 kinase activity to inflammatory syndromes in human and whether RIPK1 kinase inhibition represents a valid therapeutic approach to treat inflammation-related diseases. Therefore, there is the need to elucidate the role of RIPK1 kinase activity in the execution of cell death downstream of immune receptors such as TNFR1 and TLRs in human cells. Hence, the notion that RIPK1 is not involved in apoptosis upon IKK blockade is of general interest for the entire cell death and inflammation field. In addition, the evidence provided in this study suggest that RIPK1 inhibition would not interfere with the ability of the host to clear Yersinia.

Overall, the experiments are well performed, with the most appropriate controls. The results are clearly presented and conclusions justified. However, some additional experiments would be required to strengthen the mechanistic aspect of the study.

Major comment #1: *The authors clearly showed that in hMDM Yersinia- and LPS/IKKi-induced apoptosis is RIPK1 independent and Caspase-8-dependent. Accordingly, they observed cleaved Caspase-3. In absence of RIPK1, the only known DD-containing protein that can nucleate FADD and Caspase-8 for this last to get activated is TRADD. The authors should compare RIPK1 and TRADD expression between mBMDMs and hMDM to role in or out the possibility that the difference between human and mouse is due to the different expression levels of RIPK1 and TRADD. Once can indeed envisage the scenario where in human macrophages, unlike mouse cells, TRADD is significantly more abundant than RIPK1 and therefore it is the main molecule responsible for Caspase-8 activation. Furthermore, the authors should address whether TRADD deletion or downregulation blocks/attenuate IKK-inhibition mediated death.*

> **Response:** We thank the Reviewer for their insightful suggestion. Indeed, we considered a potential role for TRADD as an adaptor molecule in human cells instead of RIPK1. In response to the Reviewer's suggestion to compare RIPK1 and TRADD expression between murine BMDMs, hMDMs, and THP-1 macrophages, we observed comparable levels of RIPK1 across all cell types. Additionally, we observed virtually no detectable TRADD in BMDMs using a cross-species-reactive TRADD antibody, in contrast to primary

hMDMs and THP-1 cells, consistent with the Reviewer's idea that different adaptors may be differentially expressed. These data are now provided in **Figure S3F**. However, despite the high levels of TRADD expression in human macrophages, we found that CRISPR-mediated deletion of TRADD or treatment with the small molecule TRADD inhibitor, Apostatin-1 (Apt-1), had no effect on caspase-8 activation and cell death. These data are now provided in **Figure 3E, F**.

Major comment #2: *Yersinia infection and LPS/IKKi treatments also induce Caspase-9 activation. The authors should determine the contribution Caspase-9 activation to the apoptotic phenotype, for example by deleting/downregulating Bid or by over-expressing Bcl-2.*

Response: We thank the Reviewer for their questions regarding the contribution of caspase-9 to apoptosis in human macrophages. To address this question, we utilized siRNA-mediated silencing of Bid in THP-1 macrophages, as suggested by the Reviewer. Following LPS+IKKi treatment, we observed no significant decrease in cell death despite substantial Bid knockdown. These data are now provided in **Figure S2F, G**. Altogether, these data indicate that while the intrinsic apoptosis pathway is activated following IKK blockade, it is likely not required to execute cell death. However, it possible that due to incomplete siRNA-mediated knockdown, complete genetic deletion of Bid using CRISPR/Cas9 would impact cell death in human macrophages following LPS+IKKi treatment. We plan to directly test this hypothesis in future studies.

Major comment #3: *In figure 4 it would be useful to have the cleaved-Caspase-8 and cleaved-Caspase-9 immunoblotting for the cFLIP overexpressing cells infected with Yersinia or treated with LPS/IKKi. This would indicate whether the rescue of the apoptotic phenotype is partial only because of incomplete inhibition of Caspase-8 by cFLIP.*

Response: We thank the Reviewer for this important question. We observed a reduction in both caspase-8 and caspase-9 cleavage in the cFLIP-overexpressing cells, but there were still detectable levels of caspase cleavage, particularly of caspase-9, indicating incomplete block of caspase cleavage by cFLIP. These data are now provided in **Figure S4F**.

Major comment #4: *In figure 5, the cleaved-Caspase-8 immunoblotting should be added for all the 4 cell lines.*

Response: We thank the Reviewer for their suggestion. We now observe by immunoblot in hMDMs, Jurkat cells, and PANC-1 cells that caspase-8 is cleaved upon TNF+IKKi treatment, and that Nec-1 treatment does not block caspase-8 activation. Furthermore, our data demonstrate that TNF+SMAC Mimetic (TNF+SM) treatment induced RIPK1 activity-*dependent* death in Jurkat cells and PANC-1 cells. Critically, we observed reduced caspase-8 cleavage in Jurkat cells and PANC-1 cells with Nec-1 treatment. These new data are provided in **Figures S5A–C**.

Reviewer #2:

Overall summary: *The study of Neha Nataraj and colleagues concerns a potentially important issue. By applying Yersinia enterocolitica as tool for mediating infection-related cell death the role of IKK in controlling the cytotoxic activity of RIPK1 was investigated in human cells. Yersinia is well characterized to induce apoptosis in murine macrophages and the exploration of the mechanisms involved had previously provided valuable new insights into the regulation of cellular life and death during infection. However, it is less clear whether the findings obtained on murine*

cells correspond well with human cell infection. Surprisingly, the current work now indicates that RIPK1, which has been identified as critical control element in the cell death modalities of mouse cells, is dispensable for Yersinia-induced death of human macrophages. It is thus suggested that RIPK1 may fulfil distinct tasks in human cell biology which could potentially be relevant for therapeutic targeting of RIPK1 in human diseases. The study addresses a timely and interesting question. There are, however, some conceptual and methodological concerns that should be specified to substantiate the conclusions of the study.

Major comment #1: *The time courses of the investigated events require more precise synchronization. It seems that Yersinia- / IKK inhibition-induced cell death starts already at around 5 to 7 h (Fig. 1C, 2A, 2B, S1D). However, processing of caspase-8 and caspase-3 was investigated at much later time points (18 h). As caspase-8 is supposed to be the upstream initiator caspase it should be analysed whether capase-8 activation precedes onset of apoptosis and processing of caspase-9 (5 h, Fig S1D).*

> **Response:** We thank the Reviewer for this comment. We apologize, as we realized that we made a typo and mistakenly noted in the figure caption that lysates probed for caspase-8 were taken at an 18 hour timepoint. In actuality, these lysates were taken at a 5 hour timepoint, and we have now corrected the figure caption. To address the Reviewer's suggestion, we have now analyzed caspase-8 and caspase-9 cleavage over a 1 to 4 hour time-course in hMDMs. We found that processing of both caspase-8 and caspase-9 can be detected beginning at 2 hours post-stimulation/infection. These new data are provided in **Figure 2G**.

Major comment #2: *Similarly, the role of RIPK1 investigated in Fig. 3 should be evaluated also in the early phase of cell death initiation. At the late time points in Fig. 3 the cells may already have switched to RIPK1-independent cell death modes and a potential involvement of RIPK1 in cell death initiation may have been missed.*

> **Response:** We appreciate the Reviewer's point. Thus, we performed extensive time-courses for LDH release and kinetic analysis of PI uptake in THP-1 macrophages. Notably, and consistent with other work in the field, THP-1 macrophages are quite resistant to cell death relative to primary cells, and we do not observe detectable cell death in THP-1 cells until later timepoints, as measured by LDH release or PI uptake. As shown below (**Reviewer Figure 1**), WT THP-1 cells did not undergo IKKi-mediated cell death above the

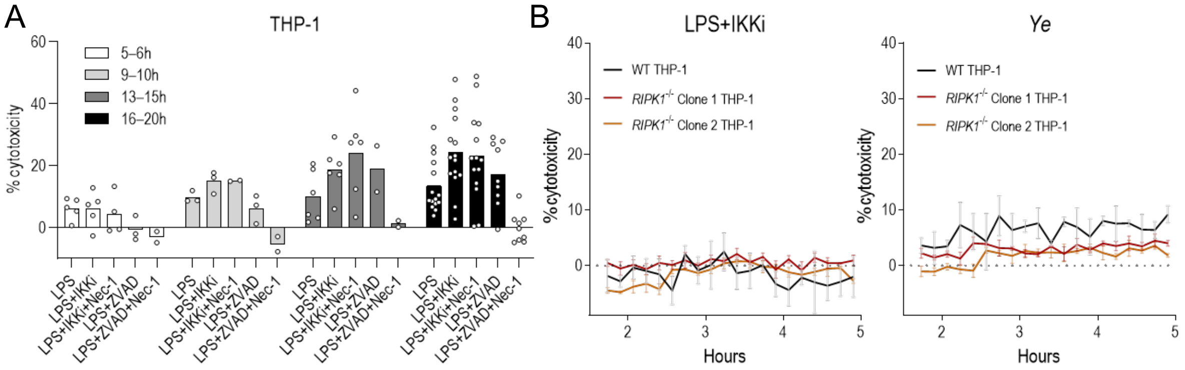

Reviewer Figure 1. (A) THP-1 cells were pre-treated with IKKi ± Nec-1 or ZVAD ± Nec-1 for 1 h and stimulated with LPS. Cytotoxicity was measured by LDH release at the indicated timepoints. Each data point represents an independent experiment. **(B)** WT and *RIPK1$^{-/-}$* THP-1 cells were pre-treated with IKKi and stimulated with LPS or infected with *Y. enterocolitica*. Cytotoxicity was measured by PI uptake over 5 h. Representative of 3 independent experiments.

LPS control in response to LPS+IKKi until after 10 hours post-stimulation, and at no point do we observe RIPK1 activity-dependent cytotoxicity following LPS+IKKi treatment, in contrast to LPS+ZVAD treatment.

We also examined caspase-3 cleavage by immunoblot in THP-1 cells treated with LPS+IKKi or infected with *Y. enterocolitica* for 5 hours. We detected low levels of caspase-3 cleavage, which was not blocked with Nec-1 treatment, indicating that RIPK1 activity is dispensable at earlier timepoints (**Reviewer Figure 2**). Additionally, we observed comparable levels of caspase-3 processing in WT and *RIPK1*$^{-/-}$ THP-1 cells at 5 hours, indicating that apoptosis at earlier timepoints is also RIPK1-independent in human macrophages. This new data is provided in **Figure S3E**.

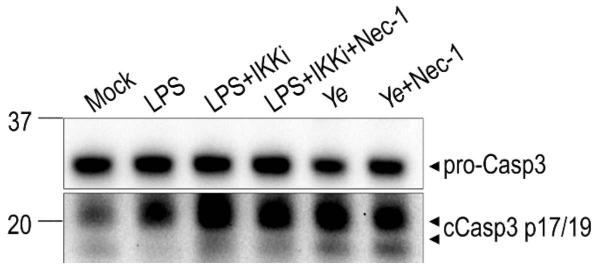

Reviewer Figure 2. THP-1 cells were pre-treated with IKKi or Nec-1 for 1 h and stimulated with LPS or infected with *Y. enterocolitica* for 5 h. Representative of 2–3 independent experiments.

Major comment #3: *In the same line, the phosphorylation of RIPK1 should be assessed at early time points, as the RIPK1 kinase activity and phosphorylation is supposed to be relevant for the initiation of cell death. It does not become clear from the manuscript at which time point RIPK1 phosphorylation was analysed in Fig. 2D. Fig. 2D and 2E are not correctly assigned in the figure caption.*

Response: We thank the Reviewer for this comment. We apologize for our oversight in not indicating the timepoint in the figure caption for this western blot, which was 6 hours post-stimulation. We have also corrected the **Figure 2** caption. To address the Reviewer's comment regarding early timepoints, we have included a time-course of RIPK1 phosphorylation in hMDMs. We detect robust phosphorylation at 1 hour post-stimulation with LPS+ZVAD treatment. Notably, we do not observe phosphorylation of RIPK1 in response to LPS+IKKi treatment at 1, 2, or 3 hours post-stimulation. These data are now provided in **Figure S2B**.

Minor comment #1: *As the study indicates that RIPK1 is dispensable for Yersinia- / IKK inhibition-induced apoptosis in human macrophages it could be worth to address experimentally whether the cell death-related, so called "ripoptosome", known from mouse cell death models, forms in human cells in the investigated conditions.*

Response: We thank the Reviewer for their suggestion. We agree that it would be interesting to characterize the nature of the complex formed in human cells, given that RIPK1 and RIPK1 kinases activity are dispensable for apoptosis in response to *Yersinia* or IKK blockade. It is currently unclear whether this complex is a bona fide Ripoptosome, given the lack of role of RIPK1. However, to test whether other known components of this complex might play a role, we investigated the potential role of FADD in a RIPK1-independent complex, as FADD contributes both to stabilization of caspase-8:cFLIP heterodimers, which block both apoptosis and necroptosis, and contributes to caspase-8-dependent apoptosis. We therefore generated *FADD*$^{-/-}$ THP-1 single-cell clones using

CRISPR/Cas9 (**Figure S2I, J**). Critically, in contrast to *RIPK1*$^{-/-}$ THP-1 cells, LPS stimulation alone induced cell death in *FADD*$^{-/-}$ cells (**Figure S2K**), and this cell death was blocked by either Nec-1 (RIPK1 inhibitor) or with GSK'872 (RIPK3 inhibitor). These data indicate that necroptosis is responsible for LPS-induced cell death in *FADD*$^{-/-}$ cells. Thus, upon LPS stimulation of human macrophages, FADD protects cells from undergoing necroptosis, similar to its role in murine cells, and likely due to the role of FADD in stabilizing caspase-8:cFLIP heterodimers to promote their pro-survival/anti-necroptosis function. Importantly, *FADD*$^{-/-}$ cells also undergo significantly less apoptosis in response to LPS+IKKi treatment (**Figure S2L**) and significantly less caspase-3/7 activation following *Yersinia* infection (**Figure S2M**). Thus, FADD appears to be largely required for this cell-extrinsic apoptosis pathway in human macrophages. We are very interested in continuing our studies by immunoprecipitating FADD and caspase-8 in human macrophages to test if a cell death complex forms in the presence or absence of RIPK1, and if so, what its composition is, which we will pursue in future studies.

Minor comment #2: *The results shown in Fig. 4 suggest that downregulation of cFLIP-L expression is a major event responsible for Yersinia- / IKK inhibition-mediated apoptosis. To substantiate a possible coherence between protein synthesis blockade and initiation of cell death following Yersinia / LPS / TNF stimulation the data obtained with the cFLIP-L-knockout and -overexpressing cells could be complemented by experiments using cycloheximide instead of IKKi as protein synthesis inhibitor. Fig. 4C, which displays caspase activities, has an incorrect figure caption.*

Response: We thank the Reviewer for this suggestion and for noting the incorrect figure caption, which we have now corrected. In order to further substantiate the hypothesis that *Yersinia* YopP and IKK inhibitor mediate apoptosis in human macrophages by preventing upregulation of pro-survival genes such as cFLIP, we conducted a parallel experiment in hMDMs treated side-by-side with LPS+IKKi or LPS+Cycloheximide (Chx), as suggested by the Reviewer. Both treatments induced comparable levels of cell death as measured by LDH release, and neither condition was protected from cell death with RIPK1 inhibition. We also observed similarly rapid depletion of cFLIP following either treatment. These new data are now provided in **Figure S4A**. Furthermore, *CFLAR*$^{-/-}$ THP-1 cells stimulated with LPS underwent comparable levels of cell death to WT THP-1 cells treated with LPS+IKKi or LPS+Chx (**Reviewer Figure 3**), which is consistent with our previous finding that IKKi-induced downregulation of cFLIP sensitizes THP-1 cells to death similarly to cells genetically lacking cFLIP.

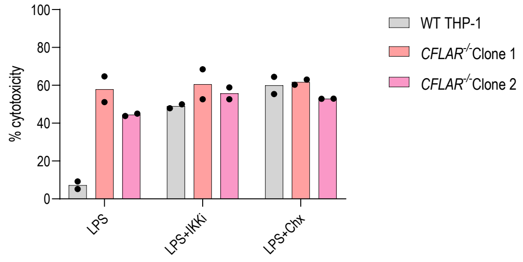

Reviewer Figure 3. WT and *CFLAR*$^{-/-}$ THP-1 macrophages were pre-treated with IKKi or Chx for 1 h and stimulated with LPS. Cytotoxicity was measured by LDH release 20–24 h post-stimulation. N=2 independent experiments.

Minor comment #3: *It is concluded from Fig. 5 that IKK blockade alone does not release the cytotoxic activity of RIPK1 in human cells. As it is known that other kinases besides IKK may prevent RIPK1 from signalling cell death it could be useful to analyse a TAK1 inhibitor in the*

experiments of Fig. 5. TAK1 acts upstream of IKK and controls additional survival-promoting signals that may be more important for controlling RIPK1 in human cells than in mouse cells. The inhibition of TAK1 would further more closely resemble the cellular YopP/YopJ effects than IKK blockade alone.

> **Response:** We thank the Reviewer for suggesting the use of a TAK1 inhibitor (TAK1i), as TAK1 is an important kinase in this complex and is also targeted by *Yersinia* YopP. As with IKK inhibition, TAK1i+LPS induced apoptosis in hMDMs, which was not significantly blocked with Nec-1. These data are now provided in **Figure S2A**.

<u>Minor comment #4:</u> *Is there an explanation why a high MOI may apparently impede Yersinia in triggering cell death (Fig. S1B)?*

> **Response:** We thank the Reviewer for their question. We have found that when macrophages are primed with LPS prior to infection with *Yersinia*, they are resistant to cell death, likely due to TLR4-dependent upregulation of pro-survival factors such as cFLIP or Bcl_{XL}. Thus, we hypothesize that the high density of bacteria results in rapid LPS stimulation of macrophages prior to YopP injection, leading to protection from cell death. This is certainly an interesting point worth further study, and we will examine this question in the future.

<u>Minor comment #5:</u> *The title suggests that blockade of IKK signalling in general mediates RIPK1-independent apoptosis in human cells. However, this has been formally addressed only for THP-1 cells by using Nec-1 and RIPK1-knockout cells. The experiments on the other human cell types solely rely on Nec-1 application which does not rule out that presence of RIPK1 by itself may be required for cell death induction independently from its kinase activity.*

> **Response:** We thank the Reviewer for this important point, and we have changed the title of our manuscript accordingly to emphasize our findings in human macrophages: "Blockade of IKK signaling induces RIPK1-independent apoptosis in human macrophages."

Reviewer #3:

<u>Overall summary:</u> *The manuscript by N. Nataraj et al. addresses species-specific differences in host response to Yersinia infection, particularly effector-mediated arrest imposed by bacteria on the inflammatory response in macrophages. Key differences in host response between humans and mice need to be explored, which is a very interesting question thus making significant the presented studies. The rationale is provided by their own observations as well as from reports by other groups. Instrumental in this paper Y. enterocolitica species of bacteria that disrupts inflammatory signaling via inhibition of IKK-complex. In summary, my impression of the novelty and significance is very positive.*

<u>Major comment #1:</u> *I wish there were more data in characterization of RIPK1-deficient THP1 cell line, which is my major concern. In short, I would like to see figure 2 to be done in RIPK1-deficient cells. Another major concern is lack of any characterization (or attempt to characterize) of what would be the alternative complex responsible for activation of caspase 8. To start with RIPK1-KO cells, caspase 8 is inactive in unstimulated cells. If it is not complexed with RIPK1, it must be bound to cFLIP? Does FADD get involved with caspase 8 in stimulated cells? For instance, caspase 8 could be pulled down and examined on the presence of FADD or cFLIP. Similarly, in kinase inhibited conditions (Figure 2), does RIPK1 bind caspase 8? As one of the ways to improve*

the manuscript, I would recommend confining it to the knockout instead of the kinase inactive conditions, which would release some space thus making the story more focused.

Response: We thank the Reviewer for their suggestions. To better characterize *RIPK1*$^{-/-}$ THP-1 cells, we examined caspase-3 cleavage by immunoblot and observed comparable levels of caspase-3 cleavage in WT and *RIPK1*$^{-/-}$ cells. These data are now provided in **Figure S3E** (see also response above to Reviewer 2).

We agree that it would be important to next characterize the cell death complex that activates caspase-8 in the absence of RIPK1. To begin to address the role of FADD, we have therefore included data with *FADD*$^{-/-}$ THP-1 cells (**Figure S2I–M**). As indicated above in our response to Reviewer 2, LPS stimulation alone induces cell death in *FADD*$^{-/-}$ cells (**Figure S2K**). Either Nec-1 treatment to block RIPK1 activity or GSK'872 treatment to block RIPK3 activity significantly reduced cell death, indicating that the LPS-induced cell death observed in the absence of FADD is necroptosis. Thus, upon LPS stimulation of human macrophages, FADD protects cells from undergoing necroptosis, likely due to the role of FADD in stabilizing caspase-8:cFLIP heterodimers to promote their pro-survival/anti-necroptosis function. Importantly, as noted above in response to Reviewer 2, *FADD*$^{-/-}$ cells also undergo significantly less cell death following LPS+IKKi treatment (**Figure S2L**) and significantly less caspase-3/7 activation following *Yersinia* infection (**Figure S2M**). Thus, FADD appears to be largely required for this apoptosis pathway in human macrophages. We are currently working on characterizing this complex by immunoprecipitating FADD and/or caspase-8 in human macrophages to determine whether a cell death signaling complex forms in WT and *RIPK1*$^{-/-}$ cells and if so, what its composition is and how it is regulated. We expect that these studies will take substantial effort and hope to include future findings in a follow-up study.

<u>Minor comment #1:</u> there are several minor issues left to be addressed after the major issues are addressed.

Response: We thank the Reviewer, and we have addressed the minor issues noted above by Reviewers 1 and 2.

We thank the editor and Reviewers for their constructive comments and suggestions on our studies. We appreciate the opportunity to revise this manuscript and believe that the new experiments we have performed and data that we have provided in response to the Reviewers' comments significantly strengthen the manuscript and the conclusions. Altogether, the revised manuscript contains 4 new main figure panels, and 18 new supplementary data Figure panels, along with corresponding changes to the text to describe and discuss these new findings. We are grateful for your consideration of our study and hope that the revised manuscript is considered appropriate for publication in *PLOS Pathogens*.
