# Supplementary material for: Blockade of IKK signaling induces RIPK1-independent apoptosis in human macrophages
Source: PLoS Pathog. 2024 Aug 26;20(8):e1012469. doi: 10.1371/journal.ppat.1012469 (PMC11407650; doi:10.1371/journal.ppat.1012469)
Supplement: Attachment — Submitted filename: Response to Reviewers.pdf [file ppat.1012469.s008.pdf]

Response to Reviewers
Thank you for the opportunity to submit our revised manuscript: "Blockade of IKK signaling induces RIPK1-independent apoptosis in human macrophages" (Manuscript ID: PPATHOGENS-D-23-01101R2). We greatly appreciate the Reviewers' evaluation of our revised submission. To address the comments made by the Reviewers, we have provided additional discussion and made corrections.

Reviewer #1:
Overall summary: *Over the past decade, mouse studies have highlighted the role of RIPK1 kinase activity in a number of cell death-driven inflammatory pathologies, including Yersinia infection, Sharpin mutant-driven dermatitis and TNF-driven lethal sepsis. However, it is still not clear the contribution of RIPK1 kinase activity to inflammatory syndromes in human and whether RIPK1 kinase inhibition represents a valid therapeutic approach to treat inflammation-related diseases. Therefore, there is the need to elucidate the role of RIPK1 kinase activity in the execution of cell death downstream of immune receptors such as TNFR1 and TLRs in human cells. Hence, the notion that RIPK1 is not involved in apoptosis upon IKK blockade is of general interest for the entire cell death and inflammation field. In addition, the evidence provided in this study suggest that RIPK1 inhibition would not interfere with the ability of the host to clear Yersinia.*

Major comment #1: *The authors addressed experimentally all my concerns and points of criticism and significantly improved the quality of the study compared to the first submission.*

> **Response:** We thank the Reviewer for their previous suggestions and review of our revised manuscript.

Reviewer #2:
Overall summary: *Neha Nataraj and colleagues resubmitted a revised manuscript of their study on the role of RIPK1 in mediating human macrophage cell death following Yersinia infection. The authors have addressed the comments previously raised by the reviewers and consequently provided additional data and information. This increased the plausibility and significance of the work. I thus feel that all major and relevant issues were convincingly resolved.*

Minor comment #1: *Fig. S2B indicates that RIPK1 is rapidly processed and cleaved by caspases following LPS and IKKi treatment. Did the authors consider that the independency of the described death of human macrophages from RIPK1 may result from rapid RIPK1 degradation, consequently entailing RIPK1-independent cell death?*

> **Response:** We thank the Reviewer for their question. RIPK1 cleavage by caspase-8 is an established hallmark of RIPK1-dependent cell-extrinsic apoptosis, and cleavage of RIPK1 by caspase-8 is a necessary mechanism to deactivate RIPK1, preventing necroptosis and unrestrained inflammation (Newton et al., Nature 2019; Lalaoui et al., Nature 2020; Tao et al., Nature 2020). We have now mentioned this detail in our manuscript as well.

Minor comment #2: *Are the authors sure that the TRADD antibody applied in Fig. S3F is cross-species-reactive to mouse and human as proposed in the comment to point #1 of reviewer one? The antibody listed in Table 2 seems to be produced from human TRADD and specificity is stated just for human TRADD, according to the information provided by the manufacturer.*

> **Response:** We thank the Reviewer for their acute attention to detail. Mistakenly, we noted the incorrect antibody in **Table 2**. We actually used a polyclonal antibody from Cell

Signaling Technology, Catalog #3694, which recognizes human, mouse, rat, and monkey (https://www.cellsignal.com/products/primary-antibodies/tradd-antibody/3694). This has now been corrected in the revised manuscript.

Reviewer #3:

Overall summary: *This is a revised submission of the manuscript by by Dr. Igor E Brodsky and colleagues, which fully addressed all the concerns raised in the initial submission. I have no further question and enthusiastically recommend its acceptance.*

Response: We thank the Reviewer for their previous suggestions and review of our revised manuscript.

We thank the editor and Reviewers for their review and suggestions on our studies. We are grateful for your consideration of our study and hope that the revised manuscript is considered appropriate for publication in *PLOS Pathogens*.
